# The obesity paradox in early and advanced HER2 positive breast cancer: pooled analysis of clinical trial data

**DOI:** 10.1038/s41523-021-00241-9

**Published:** 2021-03-22

**Authors:** Natansh D. Modi, Jin Quan Eugene Tan, Andrew Rowland, Bogda Koczwara, Ahmad Y. Abuhelwa, Ganessan Kichenadasse, Ross A. McKinnon, Michael D. Wiese, Michael J. Sorich, Ashley M. Hopkins

**Affiliations:** 1 College of Medicine and Public Health, Flinders University, Adelaide, SA Australia; 2 Department of Medical Oncology, Flinders Medical Centre, Adelaide, SA Australia; 3 Clinical and Health Sciences and Health and Biomedical Innovation, University of South Australia, Adelaide, SA Australia

**Keywords:** Breast cancer, Cancer epidemiology

## Abstract

While many studies have evaluated the relationship between BMI and breast cancer outcomes, it is unclear whether this relationship is consistent between early breast cancer (BC) and advanced BC. The study included 5099 patients with HER2 positive early BC (EBC) and 3496 with HER2 positive advanced BC (ABC). In the EBC cohort, higher BMI was associated with worse overall survival (OS) (HR [95% CI]: overweight = 1.30 [1.13–1.51]; obese = 1.37 [1.14–1.64], *P* = < 0.001), and worse disease-free survival (overweight = 1.10 [0.98–1.24]; obese = 1.20 [1.04–1.39], *P* = 0.061). In contrast, for the ABC cohort, higher BMI was significantly associated with improved OS (overweight = 0.85 [0.76–0.96]; obese = 0.82 [0.72–0.95], *P* = 0.014), and progression-free survival (overweight = 0.91 [0.83–1.01]; obese = 0.87 [0.77–0.98], *P* = 0.034). In this large high-quality dataset, higher BMI was independently associated with worse survival in EBC, paradoxically in ABC higher BMI was independently associated with improved survival.

## Introduction

Higher body mass index (BMI) is associated with an increased risk of developing many types of cancer including human epidermal growth receptor 2 (HER2) positive breast cancer (BC)^1,2^. Presumably, this is a result of elevated levels of circulating sex hormones (e.g., estrogen, estrone, and testosterone), high serum leptin, and chronic inflammation that are associated with high BMI and high adiposity, which contribute to an increased risk of developing BC^3,4^. Recent evidence also demonstrates high BMI as prognostic of poor outcomes in patients with early breast cancer (EBC), with the finding most established for hormone receptor-positive and pre-/peri-/early postmenopausal cohorts^4–21^. However, heterogeneity in the BMI–EBC survival relationship has been observed between the BC subtypes and therapies^6,15^. Thus, while evidence suggests high BMI is likely associated with a poor outcome in patients with HER2 positive EBC^9,12,13^, confirmation using high-quality data from a population treated with contemporary therapy is required^7,15^.

Importantly, there is current interest in exploring the “obesity paradox” in advanced cancers^22,23^, where elevated BMI is associated with improved survival compared to normal BMI^24,25^. Where the BMI–survival relationship has been explored in advanced BC (ABC), the sample has generally been small (i.e., *n* < 800) and observational^26–34^. Further to this, most studies have been nonspecific regarding early versus ABC, ABC subtypes (e.g., HER2 positive disease), and/or treatments used^26–35^. Coincidingly, results have been conflicting with respect to whether a paradox exists in ABC^30,32^, some studies showing no association with BMI^30,31,34,35^, and others finding that higher BMI is associated with poorer outcomes^26–29^. Owing to these conflicts^26,30–32^, as well as the known heterogeneity in the association between BMI and survival according to BC subtypes and treatment in EBC^6^, there is a need to establish the relationship between BMI and survival in HER2 positive ABC and HER2 positive EBC in patients receiving contemporary treatment options.

This study, therefore, aimed to determine the association between BMI and survival outcomes according to HER2 positive BC status (early vs. advanced).

## Results

### Association between BMI and survival outcomes in HER2 positive EBC

Data was available for 5099 HER2 positive EBC patients, of which 102 (2%) were underweight, 2433 (48%) normal weight, 1689 (33%) overweight, and 836 (16%) obese (Supplementary Table 1). Median follow-up was 132 months [95% CI: 132–132] in HERA.

**Fig. 1:**
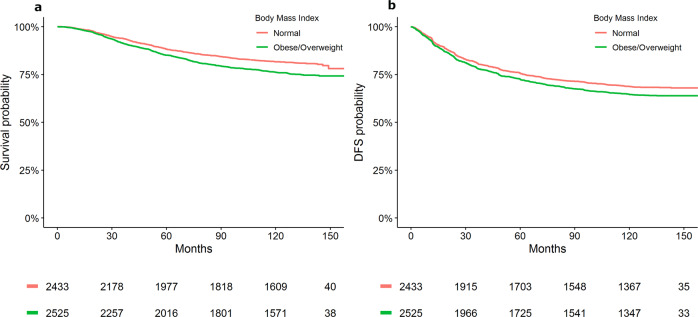
Kaplan–Meier plot representing survival outcomes by BMI status in early breast cancer [Data from HERA study]. **a** Overall survival; **b** disease-free survival.

In the HER2 positive EBC cohort, overweight and obese BMI were significantly associated with worse OS (HR [95% CI]: overweight = 1.30 [1.13–1.51]; obese = 1.37 [1.14–1.64]; underweight = 0.80 [0.47–1.37]; *P* = < 0.001). For DFS, the BMI association did not reach statistical significance (overweight = 1.10 [0.98–1.24]; obese = 1.20 [1.04–1.39]; underweight = 0.92 [0.63–1.36]; *P* = 0.061) (Table 1). Supplementary Table 2 outlines univariable analysis describing the association between BMI and survival outcomes. Figure 1 presents Kaplan–Meier estimates of OS and DFS by overweight/obese versus normal BMI category.

**Table 1:** Adjusted analysis of pretreatment body mass index with survival outcomes in EBC.

	Overall survival			Disease-free survival	
	*N*	HR [95% CI]	*P*	HR [95% CI]	*P*
BMI—WHO classification^a^			<0.001		0.061
Normal	2207	1		1	
Obese	761	1.37 [1.14–1.64]		1.20 [1.04–1.39]	
Overweight	1519	1.30 [1.13–1.51]		1.10 [0.98–1.24]	
Underweight	98	0.80 [0.47–1.37]		0.92 [0.63–1.36]	

*CI* Confidence interval, *HR* hazard ratio, *N* number of subjects, *ECOG PS* Eastern cooperative oncology group performance status, *BMI* body mass index, *WHO* World Health Organization, *ER/PR* estrogen receptor/progesterone receptor.

^a^Adjustment variables: age, race, histology grade, ECOG PS, ER/PR status, diabetes, and cardiovascular comorbidities.

No significant heterogeneity in effect size of BMI was apparent between the treatment arms of HERA for OS or DFS (Supplementary Table 3). No significant interactions between BMI category and ER/PR status were identified for OS or DFS (Supplementary Table 12, Supplementary Figs. 1, 2).

### Association between BMI and survival outcomes in HER2 positive ABC

Data was available for 3496 HER2 positive ABC patients, of which 100 (3%) were underweight, 1508 (43%) normal weight, 1060 (30%) overweight, and 778 (22%) obese (Supplementary Table 4). Median follow-up was 50 months [95% CI: 49–51] in CLEOPATRA, 35 months [34–36] in MARIANNE, 47 months [46–49] in EMILIA, and 35 months [34–36] in TH3RESA.

**Fig. 2:**
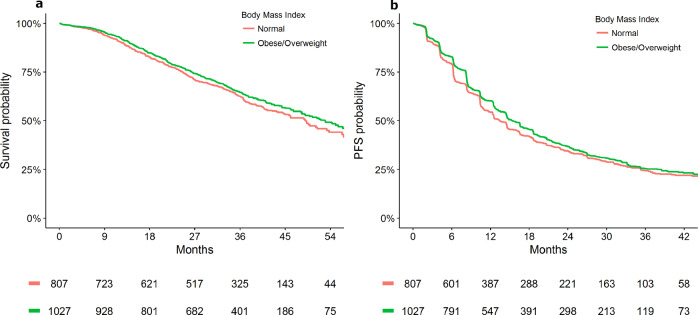
Kaplan–Meier plot representing survival outcomes in the first-line therapy cohort by BMI status in advanced breast cancer [Data from CLEOPATRA and MARIANNE study]. **a** Overall survival; **b** progression-free survival.

**Fig. 3:**
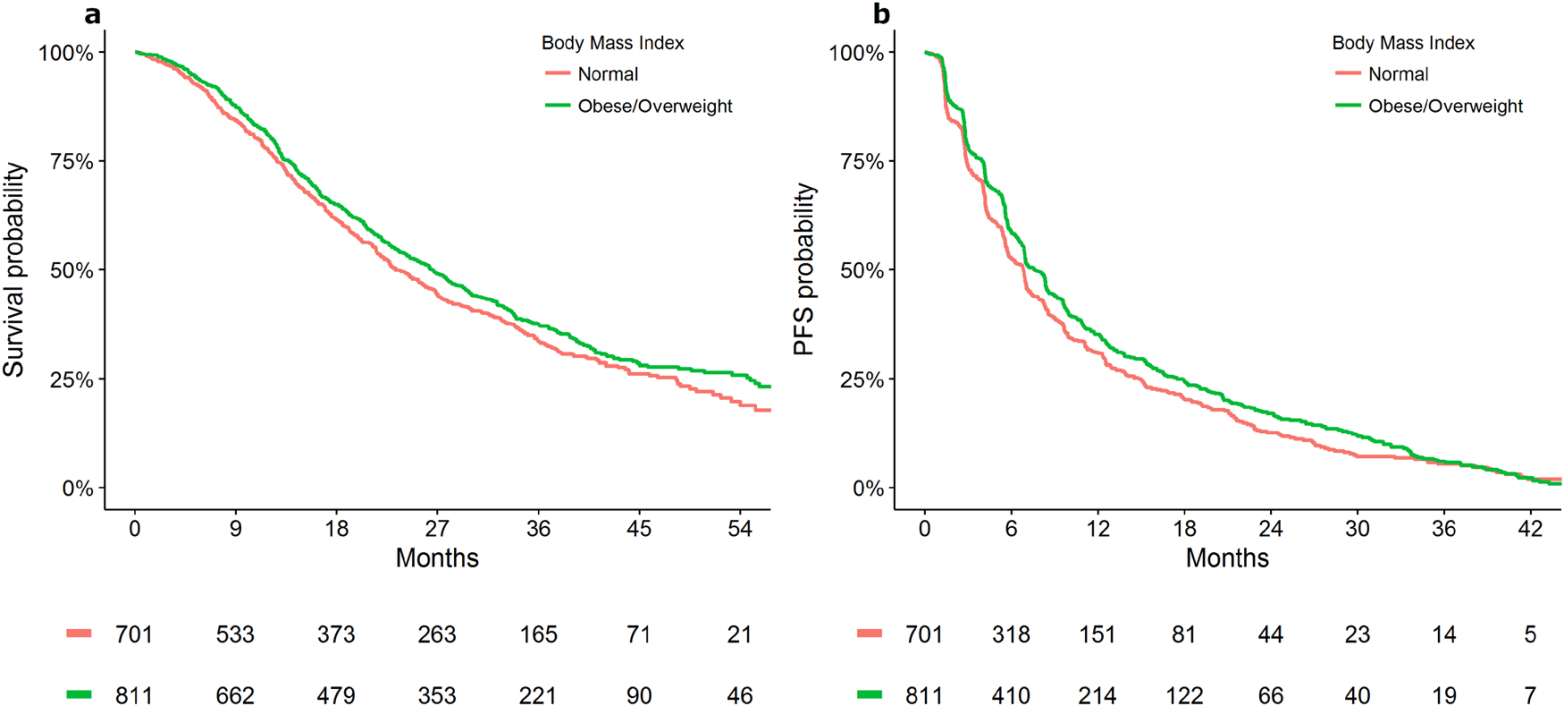
Kaplan–Meier plot representing survival outcomes in the later-line therapy cohort by BMI status in advanced breast cancer [Data from EMILIA and TH3RESA study]. **a** Overall survival; **b** progression-free survival.

In the HER2 positive ABC cohort, overweight and obese were significantly associated with improved OS compared to those with normal BMI (HR [95% CI]: overweight = 0.85 [0.76–0.96]; obese = 0.82 [0.72–0.95]; underweight = 1.02 [0.76–1.37]; *P* = 0.014), and PFS (overweight = 0.91 [0.83–1.01]; obese = 0.87 [0.77–0.98]; underweight = 1.16 [0.90–1.48]; *P* = 0.034) (Table 2). Supplementary Table 5 outlines univariable analysis describing the association between BMI category and survival outcomes. Figures 2, 3 presents Kaplan–Meier estimates of OS and PFS by BMI category (overweight/obese versus normal BMI) for patients who received first-line and later-line therapies, respectively. No statistically significant heterogeneity in results was apparent between lines of therapy and (Supplementary Table 9). Sensitivity analyses for the length of follow-up and adjustment variables resulted in no meaningful differences (Supplementary Tables 10, 11). No significant heterogeneity in effect size of pretreatment BMI was apparent between studies (Supplementary Table 6). No significant interactions between BMI and ER/PR status were identified for OS or PFS (Supplementary Table 13, Supplementary Figs. 3, 4).

**Table 2:** Adjusted analysis of pretreatment body mass index with survival outcomes in ABC.

	Overall survival		Progression-free survival		
	*N*	HR [95% CI]	*P*	HR [95% CI]	*P*
BMI—WHO classification^a^			0.014		0.034
Normal	1404	1		1	
Obese	715	0.82 [0.72–0.95]		0.87 [0.77–0.98]	
Overweight	997	0.85 [0.76–0.96]		0.91 [0.83–1.01]	
Underweight	91	1.02 [0.76–1.37]		1.16 [0.90–1.48]	

*CI* Confidence interval, *HR* hazard ratio, *N* number of subjects, *ECOG PS* Eastern cooperative oncology group performance status, *BMI* body mass index, *WHO* World Health Organization, *ER/PR* estrogen receptor/progesterone receptor.

^a^Adjustment variables: age, race, albumin count, ECOG PS, ER/PR status, presence of visceral disease and brain metastasis, diabetes and cardiovascular comorbidities.

## Discussion

Pretreatment overweight/obese BMI was independently associated with worse survival outcomes in HER2 positive EBC. Conversely, pretreatment overweight/obese BMI was independently associated with improved survival outcomes in HER2 positive ABC. The results of this study describe the presence of a marked obesity paradox in HER2 positive BC, which was consistent regardless of the use of contemporary therapy or the line of therapy.

The association of high BMI with poor outcomes in HER2 positive EBC treated with contemporary therapy outlined in this analysis is consistent with literature findings in EBC regardless of subtype^4–21^.

The obesity paradox associations of overweight/obese BMI with survival outcomes in BC outlined in this analysis are consistent with previous studies that investigated the association between BMI and outcomes in other cancer types^32,36,37^. For example, a large (*n* = 2046) recent study in metastatic melanoma identified high BMI (obese) was associated with improved survival outcomes^37^, with the effect being most pronounced in males. It was hypothesized that a contributing factor may be elevated circulating oestradiol, mediated, in part, by elevated adiposity associated with high BMI^37,38^. In the present study, the importance of the obesity paradox was demonstrated in a cohort of HER2 positive ABC patients. Further, the effects of high BMI were independent and unchanged in effect size by ER/PR status, which was not assessed by a small prior study of metastatic BC patients receiving systemic palliative chemotherapy, where overweight patients had significantly improved survival^32^.

Recently, Krasniqi et al.^26^ demonstrated BMI ≥ 30 was associated with worse OS in an observational cohort of 709 HER2 positive ABC patients treated with pertuzumab, trastuzumab, and/or T‐DM1. Albeit, the BMI association with time to failure of first-line therapy was not statistically significant^26^. Further, it is unclear what categorization of participants with BMI < 18.5 occurred, and worsened OS was not apparent in the BMI of 25 to 29.9 compared to “normal weight”^26^. Nonetheless, our study, which uses a much larger pooled clinical trial cohort, demonstrated contrasting results of pretreatment overweight/obese BMI being independently associated with improved survival outcomes in HER2 positive ABC. The cause of the difference between our study and Krasniqi et al.^26^ as well as between other ABC studies indicating a paradox^30,32^, that there is no BMI association^30,31,34,35^, or that high BMI is associated with poor outcomes^26–29^ remains unknown; however, it does highlight the need for further investigation. In strength to the study herein the obesity paradox demonstrated was consistently observed across the four pooled HER2 positive ABC clinical trials (Supplementary Table 6).

Hypotheses for the obesity paradox in advanced cancers include an association between the nutritional reserve and cancer-related cachexia which is characterized by low body weight, anorexia, high ECOG PS, and low albumin^37,39^. In the present study, the association between high BMI and improved survival was independent of ECOG PS and albumin. This highlights an urgent need to investigate the underlying mechanism of the obesity paradox, as it will be important to identify whether the effect tapers off, or reverses as per Krasniqi et al.^26^ when BMI approaches severe obesity (BMI ≥ 35 kg/m^2^)^23^, and whether methods to safely increase BMI can result in improved survival^32,40^. Future research should also aim to elucidate the complex role of body composition on survival outcomes^41^. Specifically, there is a need to understand the impacts of adiposity versus lean mass versus sarcopenic obesity versus other measures of body composition (e.g., fat-free mass or fat mass). There is also a need to better understand whether the BMI/body composition associations differ between specific BC subtypes (e.g., HER2 positive/negative versus triple-negative BC, ER/PR negative versus ER/PR positive), advanced cancer types (e.g., BC versus lung cancer) and received cancer treatments. For example, while this study found a non-significant interaction for both the EBC and ABC cohorts according to ER/PR status; for the ER/PR negative EBC cohort the OS association effect sizes (HRs) were 1.25 and 1.21 for the obese and overweight groups, respectively, while for ER/PR positive the OS association effect sizes were 1.55 and 1.44. This trend towards worsened survival in EBC with ER/PR expression is like prior findings in small studies^5,42,43^. That a statistical interaction was not detected according to ER/PR status within the EBC cohort, may be an indication of the need for studies larger than that herein to detect differences by BC subtypes in either EBC or ABC.

Randomized clinical trials (RCTs) form the basis of current evidence-based medicine^44^. However, strict inclusion criteria of RCTs can limit their generalizability to the real-world settings, for example, the prevalence of obesity in the available study population (~20%) is lower than the current prevalence of obesity in US women (~40%)^45^. Nonetheless, the study pooled large high-quality data from five contemporary RCTs (HERA, CLEOPATRA, MARIANNE, TH3RESA, and EMILIA) to increase study power and generalizability. Further, the high-quality data allowed robust adjustment for many known prognostic variables which are often not available in real-world databases. Notwithstanding, the biological relationship between body composition metrics with known prognostic factors is complex and poorly elucidated—limiting the ability of this research to conclude causal impacts of BMI on survival outcomes in BC^46–48^. Thus, it is a significant strength of the study that the univariable and adjusted results presented a consistent association of an obesity paradox. Given the EBC and ABC cohorts were non-matched it is an important future question to investigate the longitudinal relationship of BMI throughout a patient’s transition from EBC to ABC. While the sample was large, the study was inadequately powered to assess the underweight (BMI ≤ 18.5 kg/m^2^) and severely obese (BMI ≥ 35 kg/m^2^) populations (Supplementary Tables 7, 8 presents preliminary evidence), and ethnic subpopulations (e.g., African Americans^49^). Owing to the significant prognostic impact of BMI demonstrated in this study, and the differences between early and advanced disease; future RCTs in HER2 positive BC may consider evaluating BMI as a potential stratification factor. It is acknowledged that the present study was unable to examine the BMI–survival association in HER2 positive ABC not treated with anti-HER2 therapy, as all patients in the analysed cohort were treated with anti-HER2 therapy.

In conclusion, high BMI was independently associated with worse survival in HER2 positive EBC and improved survival in HER2 positive ABC, demonstrating a clear obesity paradox in this BC subtype. The results have implications on trial designs and indicate a need to understand the biological basis of obesity impacts throughout HER2 positive BC.

## Methods

### Patient population

Individual participant data (IPD) from the Roche sponsored phase III clinical trials HERA [NCT00045032]^50,51^, CLEOPATRA [NCT00567190]^52–54^, MARIANNE [NCT01120184]^55,56^, EMILIA [NCT00829166]^57,58^, and TH3RESA [NCT01419197]^59,60^ were utilized in this post hoc study. Data were accessed according to Roche policy and has been made available through Vivli, Inc (www.vivli.org). Secondary analysis of anonymized IPD was exempted from review by the Southern Adelaide Local Health Network, Office for Research and Ethics as it was classified as minimal risk research.

HERA included patients with histologically confirmed EBC (i.e., completely excised, nonmetastatic invasive BC overexpressing HER2) who were randomly assigned 1:1:1 to observation, trastuzumab 1 year, or trastuzumab 2 year^50,51^.

CLEOPATRA included patients with ABC (locally recurrent, unresectable, or metastatic HER2 positive BC) that were treatment naive (excluding prior hormonal therapy) in the advanced setting. Patients were randomly assigned 1:1 to receive either receive placebo plus trastuzumab plus docetaxel, or pertuzumab plus trastuzumab plus docetaxel^52–54^.

MARIANNE recruited patients with HER2 positive ABC that was unresectable, progressive, or locally recurrent, or previously treatment naïve metastatic BC. Patients were randomly assigned 1:1:1 to trastuzumab plus a taxane, trastuzumab emtansine (T-DM1) plus placebo, or T-DM1 plus pertuzumab^55,56^.

EMILIA included patients with ABC (unresectable, locally advanced, or metastatic HER2 positive BC) with documented progression to prior taxane and trastuzumab treatment. Participants were randomly assigned 1:1 to either lapatinib plus capecitabine, or T-DM1^57,58^.

TH3RESA included patients with ABC (locally recurrent, unresectable, or metastatic HER2 positive BC) with documented disease progression to trastuzumab and lapatinib in the advanced setting and had received a taxane in any setting. Patients were randomly assigned 1:2 to physician’s choice treatment or T-DM1^59,60^.

### Predictors and outcomes

The primary assessed outcome was overall survival (OS), with disease-free survival (DFS), and progression-free survival (PFS) assessed as secondary outcomes. OS was defined as the time from randomization to the last follow-up or death from any cause consistent across all studies. DFS was defined in HERA as the time from randomization to the first occurrence of any of the following events: recurrence of BC at any site; development of ipsilateral or contralateral BC (including ductal carcinoma in situ but not lobular carcinoma in situ); development of second non-breast malignant disease (other than basal-cell or squamous-cell carcinoma of the skin or carcinoma in situ of the cervix); or death from any cause without documentation of a cancer-related event. PFS was defined within CLEOPATRA and EMILIA as the time from randomization to disease progression or death from any cause, with progression assessed by the investigators using the Response Evaluation Criteria in Solid Tumors (RECIST) version 1.0 (CLEOPATRA and EMILIA) or RECIST version 1.1 (TH3RESA and MARIANNE).

BMI was calculated as total body weight (kg) divided by the square of body height (m^2^)^61^. BMI was categorized according to the WHO definitions (underweight <18.5, normal 18.5–25.0, overweight 25.0–30.0, and obese >30.0 kg/m^2^).

Available pretreatment characteristic data within HERA (EBC dataset) included BMI category, race (Asian, white, black or African American), histology grade, estrogen/progesterone receptor status (ER/PR), presence of cardiovascular disease (CVD), diabetes mellitus (DM), and Eastern Cooperative Oncology Group performance status (ECOG PS).

Available pretreatment characteristic data within EMILIA, TH3RESA, MARIANNE, and CLEOPATRA (ABC dataset) included BMI category, age, race (Asian or Non-Asian), presence of brain metastasis and visceral disease, albumin below the lower limit of normal (<LLN), ECOG PS, ER/PR, any prior taxane, anthracycline or trastuzumab use, and presence of CVD or DM.

### Statistical analysis

Cox proportional hazard analysis was used to assess the association between pretreatment BMI category with OS and PFS. Complete case analyses were conducted. Results were reported as hazard ratios (HR) with 95% confidence intervals (95% CI). Statistical significance was set at a threshold of *P* < 0.05 and was determined via the likelihood ratio test. EBC and ABC IPD were analysed separately. All analyses were stratified by study and treatment. Primary analyses were adjusted for known confounders. The heterogeneity of BMI effect by ER/PR status was assessed using a treatment-by-biomarker interaction term.

Kaplan–Meier analysis was used for plotting and estimating OS, DFS, and PFS probabilities. All data analysis was conducted using R version 3.4.3.

### Reporting summary

Further information on research design is available in the Nature Research Reporting Summary linked to this article.

## Supplementary information

Supplementary Tables and Figures

Reporting Summary Checklist

## Data Availability

The data generated and analysed during this study are described in the following data record: 10.6084/m9.figshare.14046287^62^. No new data were generated in this study. IPD from the Roche sponsored phase III clinical trials HERA [data ID: NCT00045032], CLEOPATRA [data ID: NCT00567190], MARIANNE [data ID: NCT01120184], EMILIA [data ID: NCT00829166], and TH3RESA [data ID: NCT01419197] were utilized in a post hoc study. The IPD are available via the Center for Global Clinical Research Data’s Vivli data sharing platform: https://vivli.org/. Data can be searched via the data IDs provided above, but a request must be logged in order to access the data. Vivli has not contributed to or approved and is not in any way responsible for the contents of this publication.

## Code availability

The code for data processing and visualization is written in R and available upon request (N.M.: Natansh.modi@flinders.edu.au).
