# Supplementary material for: The obesity paradox in early and advanced HER2 positive breast cancer: pooled analysis of clinical trial data
Source: NPJ Breast Cancer. 2021 Mar 22;7:30. doi: 10.1038/s41523-021-00241-9 (PMC7985140; doi:10.1038/s41523-021-00241-9)

Supplementary Tables

Supplementary Table 1: Summary of patient characteristics by treatment arm in early breast cancer

	Total<br>No. 5,099	Placebo<br>No. 1,744	Trastuzumab 1-year<br>No. 1,682	Trastuzumab 2-years<br>No. 1,673
Actual treatment (arm)				
Placebo	1,744(34%)	1,744 (100%)	0 (0%)	0 (0%)
Trastuzumab 1-year	1,682(33%)	0 (0%)	1,682 (100%)	0 (0%)
Trastuzumab 2-years	1,673(33%)	0 (0%)	0 (0%)	1,673 (100%)
Race				
White	4,254 (83%)	1,453 (83%)	1,404 (83%)	1,397 (84%)
Asian	7 (<1%)	4 (<1%)	2 (<1%)	1 (<1%)
Black or African American	19 (<1%)	6 (<1%)	8 (<1%)	5 (<1%)
Other	819 (16%)	281 (16%)	268 (16%)	270 (16%)
ECOG PS				
0	4,683 (92%)	1,590 (91%)	1,539 (91%)	1,554 (93%)
≥1	414 (8%)	152 (9%)	143 (9%)	119 (7%)
Missing	2 (<1%)	2 (<1%)	0 (0%)	0 (0%)
BMI - WHO Classification				
Normal	2,433 (48%)	832 (48%)	791 (47%)	810 (48%)
Obese	836 (16%)	287 (16%)	279 (17%)	270 (16%)
Overweight	1,689 (33%)	571 (33%)	567 (34%)	551 (33%)
Underweight	102 (2%)	35 (2%)	34 (2%)	33 (2%)
Missing	39 (1%)	19 (1%)	11 (1%)	9 (1%)
Histology grade				
Well/Moderately differentiated	1,758 (34%)	615 (35%)	580 (34%)	563 (34%)
Poorly differentiated	3,089 (61%)	1,040 (60%)	1,020 (61%)	1,029 (62%)
Missing	252 (5%)	89 (5%)	82 (5%)	81 (5%)
Estrogen / Progesterone Receptor Status				
ER and PR positive	1,512 (30%)	475 (27%)	519 (31%)	518 (31%)
ER and PR negative	2,440 (48%)	834 (48%)	804 (48%)	802 (48%)
ER or PR negative	919 (18%)	344 (20%)	291 (17%)	284 (17%)
Missing	228 (4%)	91 (5%)	68 (4%)	69 (4%)
Cardiovascular disease at baseline	1,017(20%)	353 (20%)	348 (21%)	316 (19%)
Diabetes at baseline	144 (3%)	46 (3%)	55 (3%)	43 (3%)
Age				
≤60	4,405(86%)	1,500 (86%)	1,452 (86%)	1,453 (87%)
>60	694 (14%)	244 (14%)	230 (14%)	220 (13%)

Data are median (IQR) or number of patients (%).
CI= Confidence interval, HR= Hazard ratio, N = Number of subjects, ECOG PS= Eastern cooperative oncology group performance status, BMI = Body Mass Index, WHO = World Health Organization & ER/PR = Estrogen Receptor/Progesterone Receptor

Supplementary Table 2: Univariable analysis of pre-treatment body mass index with survival outcomes in EBC

		Overall Survival		Disease Free Survival	
	N	HR [95% CI]	P	HR [95% CI]	P
BMI - WHO classification			0.001		0.003
Normal	2433	1		1	
Obese	836	1.42 [1.20 to 1.67]		1.26 [1.10 to 1.44]	
Overweight	1689	1.31 [1.14 to 1.50]		1.12 [1.00 to 1.25]	
Underweight	102	0.76 [0.45 to 1.29]		0.85 [0.58 to 1.24]	

CI= Confidence interval, HR= Hazard ratio, N = Number of subjects, BMI = Body Mass Index, WHO = World Health Organization

Supplementary Table 3: Subset analysis by Treatment arm to evaluate the effect of BMI on survival outcomes in EBC

			Overall Survival	Disease Free Survival
ACTARM	Variable	N	HR [95% CI]	HR [95% CI]
PLACEBO	BMI - WHO classification[1]			
	Normal	744	1	1
	Obese	257	1.46 [1.08 to 1.95]	1.28 [1.01 to 1.63]
	Overweight	511	1.30 [1.03 to 1.65]	1.08 [0.89 to 1.32]
	Underweight	33	0.53 [0.20 to 1.43]	0.57 [0.27 to 1.22]
TRASTUZUMAB 1-YEAR	BMI - WHO classification[1]			
	Normal	727	1	1
	Obese	253	1.55 [1.12 to 2.15]	1.33 [1.03 to 1.73]
	Overweight	509	1.63 [1.26 to 2.11]	1.33 [1.08 to 1.64]
	Underweight	33	1.05 [0.43 to 2.58]	1.24 [0.66 to 2.36]
TRASTUZUMAB 2-YEARS	BMI - WHO classification[1]			
	Normal	736	1	1
	Obese	251	1.13 [0.81 to 1.57]	1.03 [0.79 to 1.33]
	Overweight	499	1.02 [0.78 to 1.33]	0.92 [0.75 to 1.14]
	Underweight	32	1.01 [0.41 to 2.47]	1.08 [0.57 to 2.05]
P[interaction]			0.21	0.20

1) Adjustment Variables: Age, Race, Histology grade, ECOG PS, ER/PR Status, Diabetes and Cardiovascular comorbidities.

CI= Confidence interval, HR= Hazard ratio, N = Number of subjects, ECOG PS= Eastern cooperative oncology group performance status, BMI = Body Mass Index, WHO = World Health Organization & ER/PR = Estrogen Receptor/Progesterone Receptor.

Supplementary Table 4: Summary of patient characteristics by study in advanced breast cancer

	CLEOPATRA<br>No. 808	EMILIA<br>No. 991	MARIANNE<br>No. 1,095	TH3RESA<br>No. 602
The actual treatment given				
Lapatinib + Capecitabine	0 (0%)	488 (49%)	0 (0%)	0 (0%)
Pertuzumab + Trastuzumab + Docetaxel	408 (50%)	0 (0%)	0 (0%)	0 (0%)
Pertuzumab + Trastuzumab emtansine	0 (0%)	0 (0%)	366 (33%)	0 (0%)
Physicians Choice	0 (0%)	0 (0%)	0 (0%)	184 (31%)
Placebo + Trastuzumab + Docetaxel	396 (49%)	0 (0%)	0 (0%)	0 (0%)
Placebo + Trastuzumab emtansine	0 (0%)	0 (0%)	361 (33%)	0 (0%)
Trastuzumab + Docetaxel/Paclitaxel	0 (0%)	0 (0%)	353 (32%)	0 (0%)
Trastuzumab emtansine	0 (0%)	490 (49%)	0 (0%)	403 (67%)
Missing	4 (0%)	13 (1%)	15 (1%)	15 (2%)
Study				
CLEOPATRA	808 (100%)	0 (0%)	0 (0%)	0 (0%)
EMILIA	0 (0%)	991 (100%)	0 (0%)	0 (0%)
MARIANNE	0 (0%)	0 (0%)	1,095 (100%)	0 (0%)
TH3RESA	0 (0%)	0 (0%)	0 (0%)	602 (100%)
Age				
<=60	599 (74%)	752 (76%)	795 (73%)	441 (73%)
>60	209 (26%)	239 (24%)	300 (27%)	161 (27%)
Race				
Non-Asian	546 (68%)	811 (82%)	839 (77%)	521 (87%)
Asian	261 (32%)	180 (18%)	256 (23%)	81 (13%)
Missing	1 (0%)	0 (0%)	0 (0%)	0 (0%)
BMI - WHO classification				
Normal	349 (43%)	407 (41%)	458 (42%)	294 (49%)
Obese	187 (23%)	242 (24%)	248 (23%)	101 (17%)
Overweight	241 (30%)	302 (30%)	351 (32%)	166 (28%)
Underweight	30 (4%)	23 (2%)	31 (3%)	16 (3%)
Missing	1 (0%)	17 (2%)	7 (1%)	25 (4%)
ECOG PS				
0	522 (65%)	612 (62%)	719 (66%)	262 (44%)
>=1	286 (35%)	370 (37%)	376 (34%)	338 (56%)
Missing	0 (0%)	9 (1%)	0 (0%)	2 (0%)
Brain Metastasis				
Absent	807 (100%)	913 (92%)	1,079 (99%)	529 (88%)
Present	1 (0%)	75 (8%)	14 (1%)	72 (12%)
Missing	0 (0%)	3 (0%)	2 (0%)	1 (0%)
Albumin				
< LLN	68 (8%)	82 (8%)	102 (9%)	94 (16%)
>= LLN	708 (88%)	884 (89%)	968 (88%)	487 (81%)
Missing	32 (4%)	25 (3%)	25 (2%)	21 (3%)
Visceral Disease				
Absent	178 (22%)	322 (32%)	344 (31%)	151 (25%)
Present	630 (78%)	669 (68%)	751 (69%)	451 (75%)
ER & PR Status				
ER and PR positive	224 (28%)	330 (33%)	387 (35%)	200 (33%)
ER or PR negative	157 (19%)	205 (21%)	199 (18%)	101 (17%)
ER and PR negative	408 (50%)	426 (43%)	470 (43%)	270 (45%)
Missing	19 (2%)	30 (3%)	39 (4%)	31 (5%)
Cardiovascular Disease				
Absent	581 (72%)	760 (77%)	788 (72%)	428 (71%)
Present	227 (28%)	231 (23%)	307 (28%)	174 (29%)
Diabetes				
Absent	741 (92%)	926 (93%)	997 (91%)	561 (93%)
Present	67 (8%)	65 (7%)	98 (9%)	41 (7%)

CI=confidence interval, HR=hazard ratio, BMI = Body Mass Index, WHO = World Health Organization, ER = Estrogen Receptor, PR = Progesterone Receptor, ECOG PS = Eastern Cooperative Oncology Group Performance Status, LLN = Lower Limit of Normal

Supplementary Table 5: Univariable analysis of pre-treatment body mass index with survival outcomes in advanced breast cancer

		Overall Survival		Progression Free Survival	
	N	HR [95% CI]	P	HR [95% CI]	P
BMI - WHO classification			0.165		0.044
Normal	1486	1		1	
Obese	772	0.91 [0.81 to 1.03]		0.89 [0.80 to 0.98]	
Overweight	1047	0.89 [0.80 to 1.00]		0.92 [0.84 to 1.01]	
Underweight	96	1.06 [0.79 to 1.42]		1.13 [0.89 to 1.44]	

CI= Confidence interval, HR= Hazard ratio, N = Number of subjects, BMI = Body Mass Index, WHO = World Health Organization

Supplementary Table 6: Subset analysis by STUDY to evaluate the effect of BMI on survival outcomes in advanced breast cancer

			Overall Survival	Progression Free Survival
Subgroup	Variable	N	HR [95% CI]	HR [95% CI]
CLEOPATRA	BMI - WHO classification[1]			
	Normal	324	1	1
	Obese	174	0.86 [0.63 to 1.17]	0.92 [0.72 to 1.17]
	Overweight	230	0.85 [0.65 to 1.10]	0.87 [0.71 to 1.07]
	Underweight	28	1.36 [0.78 to 2.37]	1.48 [0.95 to 2.30]
MARIANNE	BMI - WHO classification[2]			
	Normal	439	1	1
	Obese	225	0.63 [0.46 to 0.87]	0.74 [0.59 to 0.92]
	Overweight	331	0.90 [0.70 to 1.16]	0.94 [0.78 to 1.13]
	Underweight	29	1.37 [0.73 to 2.56]	1.28 [0.81 to 2.04]
TH3RESA	BMI - WHO classification[2]			
	Normal	264	1	1
	Obese	87	0.70 [0.50 to 0.98]	0.69 [0.52 to 0.94]
	Overweight	153	0.70 [0.53 to 0.92]	0.85 [0.68 to 1.07]
	Underweight	11	0.94 [0.43 to 2.05]	1.06 [0.55 to 2.03]
EMILIA	BMI - WHO classification[2]			
	Normal	377	1	1
	Obese	229	1.01 [0.82 to 1.26]	1.07 [0.87 to 1.31]
	Overweight	283	0.92 [0.75 to 1.12]	0.91 [0.76 to 1.10]
	Underweight	23	0.84 [0.49 to 1.42]	0.97 [0.59 to 1.59]
P[interaction]			0.24	0.83

1) Adjustment Variables: Age, Race, Albumin count, ECOG PS, ER/PR Status, Presence of Visceral Disease, Diabetes and Cardiovascular comorbidities.
2) Adjustment Variables: Age, Race, Albumin count, ECOG PS, ER/PR Status, Presence of Visceral Disease and Brain Metastasis, Diabetes and Cardiovascular comorbidities.

CI= Confidence interval, HR= Hazard ratio, N = Number of subjects, ECOG PS= Eastern cooperative oncology group performance status, BMI = Body Mass Index, WHO = World Health Organization & ER/PR = Estrogen Receptor/Progesterone Receptor.

Supplementary Table 7: Adjusted analysis of pre-treatment body mass index (including the severe obese subgroup) with survival outcomes in early breast cancer

		Overall Survival		Disease Free Survival	
	N	HR [95% CI]	P	HR [95% CI]	P
BMI - WHO classification[1]			<0.001		0.105
Normal	2207	1		1	
Severe Obese	214	1.24 [0.91 to 1.68]		1.26 [1.00 to 1.60]	
Obese	547	1.42 [1.16 to 1.73]		1.18 [1.00 to 1.39]	
Overweight	1519	1.30 [1.13 to 1.51]		1.10 [0.98 to 1.24]	
Underweight	98	0.80 [0.47 to 1.37]		0.92 [0.63 to 1.36]	

1) Adjustment Variables: Age, Race, Histology grade, ECOG PS, ER/PR Status, Diabetes and Cardiovascular comorbidities.

CI= Confidence interval, HR= Hazard ratio, N = Number of subjects, ECOG PS= Eastern cooperative oncology group performance status, BMI = Body Mass Index, WHO = World Health Organization & ER/PR = Estrogen Receptor/Progesterone Receptor

Supplementary Table 8: Adjusted analysis of pre-treatment body mass index (including the severe obese subgroup) with survival outcomes in advanced breast cancer

		Overall Survival		Progression Free Survival	
	N	HR [95% CI]	P	HR [95% CI]	P
BMI - WHO classification[1]			0.031		0.049
Normal	1404	1		1	
Severe Obese	238	0.81 [0.66 to 0.99]		0.82 [0.69 to 0.97]	
Obese	477	0.83 [0.71 to 0.97]		0.90 [0.79 to 1.02]	
Overweight	997	0.85 [0.76 to 0.96]		0.91 [0.83 to 1.01]	
Underweight	91	1.02 [0.76 to 1.37]		1.16 [0.90 to 1.48]	

1) Adjustment Variables: Age, Race, Albumin count, ECOG PS, ER/PR Status, Presence of Visceral Disease and Brain Metastasis, Diabetes and Cardiovascular comorbidities.

CI= Confidence interval, HR= Hazard ratio, N = Number of subjects, ECOG PS= Eastern cooperative oncology group performance status, BMI = Body Mass Index, WHO = World Health Organization & ER/PR = Estrogen Receptor/Progesterone Receptor

Supplementary Table 9: Subset analysis by line of therapy to evaluate the effect of BMI on survival outcomes in advanced breast cancer

			Overall Survival	Progression Free Survival
Subgroup	Variable	N	HR [95% CI]	HR [95% CI]
First line therapy	BMI - WHO classification[1]			
	Normal	763	1	1
	Obese	399	0.72 [0.58 to 0.90]	0.81 [0.68 to 0.95]
	Overweight	561	0.87 [0.73 to 1.04]	0.91 [0.79 to 1.04]
	Underweight	57	1.34 [0.88 to 2.03]	1.39 [1.01 to 1.91]
Later line therapy	BMI - WHO classification[1]			
	Normal	641	1	1
	Obese	316	0.90 [0.75 to 1.07]	0.93 [0.79 to 1.09]
	Overweight	436	0.83 [0.71 to 0.97]	0.90 [0.78 to 1.04]
	Underweight	34	0.85 [0.55 to 1.31]	0.95 [0.64 to 1.40]
P [interaction]			0.79	0.88

1) Adjustment Variables: Age, Race, Albumin count, ECOG PS, ER/PR Status, Presence of Visceral Disease and Brain Metastasis, Diabetes and Cardiovascular comorbidities.

CI= Confidence interval, HR= Hazard ratio, N = Number of subjects, ECOG PS= Eastern cooperative oncology group performance status, BMI = Body Mass Index, WHO = World Health Organization & ER/PR = Estrogen Receptor/Progesterone Receptor.

Supplementary Table 10: Time adjusted analysis of pre-treatment body mass index with survival outcomes in ABC

		Overall Survival		Progression Free Survival	
	N	HR [95% CI]	P	HR [95% CI]	P
BMI - WHO classification[1]			0.038		0.040
Normal	1404	1		1	
Obese	715	0.82 [0.71 to 0.96]		0.86 [0.77 to 0.97]	
Overweight	997	0.86 [0.76 to 0.98]		0.92 [0.83 to 1.01]	
Underweight	91	0.98 [0.71 to 1.36]		1.14 [0.88 to 1.46]	

1) Adjustment Variables: Age, Race, Albumin count, ECOG PS, ER/PR Status, Presence of Visceral Disease and Brain Metastasis, Diabetes and Cardiovascular comorbidities.

CI= Confidence interval, HR= Hazard ratio, N = Number of subjects, ECOG PS= Eastern cooperative oncology group performance status, BMI = Body Mass Index, WHO = World Health Organization & ER/PR = Estrogen Receptor/Progesterone Receptor

Supplementary Table 11: Adjusted analysis of pre-treatment body mass index (excluding albumin count as an adjustment variable) with survival outcomes in advanced breast cancer

		Overall Survival		Progression Free Survival	
	N	HR [95% CI]	P	HR [95% CI]	P
BMI - WHO classification[1]			0.03		0.05
Normal	1433	1		1	
Obese	733	0.85 [0.74 to 0.97]		0.88 [0.79 to 0.99]	
Overweight	1014	0.86 [0.76 to 0.96]		0.91 [0.83 to 1.01]	
Underweight	93	1.00 [0.74 to 1.35]		1.13 [0.89 to 1.45]	

Adjustment Variables: Age, Race, ECOG PS, ER/PR Status, Presence of Visceral Disease and Brain Metastasis, Diabetes and Cardiovascular comorbidities.

CI= Confidence interval, HR= Hazard ratio, N = Number of subjects, ECOG PS= Eastern cooperative oncology group performance status, BMI = Body Mass Index, WHO = World Health Organization & ER/PR = Estrogen Receptor/Progesterone Receptor

Supplementary Table 12: Subset analysis by ER/PR status to evaluate the effect of BMI on survival outcomes in EBC

		ER and PR Negative		ER or PR Positive		
Survival outcome	Variable	N	HR [95% CI]	N	HR [95% CI]	P[interaction]
Overall Survival	BMI - WHO classification[1]					0.41
	Normal	1044	1	1163	1	
	Obese	396	1.25 [0.98 to 1.59]	365	1.55 [1.18 to 2.02]	
	Overweight	803	1.21 [0.99 to 1.47]	716	1.44 [1.15 to 1.79]	
	Underweight	46	0.89 [0.44 to 1.81]	52	0.72 [0.32 to 1.63]	
Disease Free Survival	BMI - WHO classification[1]					0.47
	Normal	1044	1	1163	1	
	Obese	396	1.14 [0.94 to 1.39]	365	1.28 [1.04 to 1.58]	
	Overweight	803	1.03 [0.88 to 1.21]	716	1.19 [1.00 to 1.41]	
	Underweight	46	1.07 [0.64 to 1.79]	52	0.79 [0.44 to 1.40]	

1) Adjustment Variables: Age, Race, Histology grade, ECOG PS, Diabetes and Cardiovascular comorbidities.

CI= Confidence interval, HR= Hazard ratio, N = Number of subjects, ECOG PS= Eastern cooperative oncology group performance status, BMI = Body Mass Index, WHO = World Health Organization & ER/PR = Estrogen Receptor/Progesterone Receptor.

Supplementary Table 13: Subset analysis by ER/PR status to evaluate the effect of BMI on survival outcomes in ABC

		ER and PR Negative		ER or PR Positive		
Survival outcome	Variable	N	HR [95% CI]	N	HR [95% CI]	P[interaction]
Overall Survival	BMI - WHO classification[1]					0.93
	Normal	667	1	737	1	
	Obese	328	0.80 [0.66 to 0.98]	387	0.86 [0.71 to 1.04]	
	Overweight	457	0.85 [0.72 to 1.00]	540	0.86 [0.72 to 1.01]	
	Underweight	47	0.94 [0.61 to 1.44]	44	1.17 [0.77 to 1.78]	
Progression Free Survival	BMI - WHO classification[1]					0.44
	Normal	667	1	737	1	
	Obese	328	0.87 [0.73 to 1.03]	387	0.87 [0.74 to 1.02]	
	Overweight	457	0.89 [0.77 to 1.02]	540	0.93 [0.82 to 1.07]	
	Underweight	47	0.89 [0.64 to 1.26]	44	1.59 [1.11 to 2.27]	

1) Adjustment Variables: Age, Race, Albumin count, ECOG PS, Presence of Visceral Disease and Brain Metastasis, Diabetes and Cardiovascular comorbidities.

CI= Confidence interval, HR= Hazard ratio, N = Number of subjects, ECOG PS= Eastern cooperative oncology group performance status, BMI = Body Mass Index, WHO = World Health Organization & ER/PR = Estrogen Receptor/Progesterone Receptor.

Supplementary Figure 1: Kaplan-Meier plot representing survival outcomes by BMI status in early breast cancer ER/PR Positive Population [Data from HERA study] a) Overall survival; b) Disease-free survival

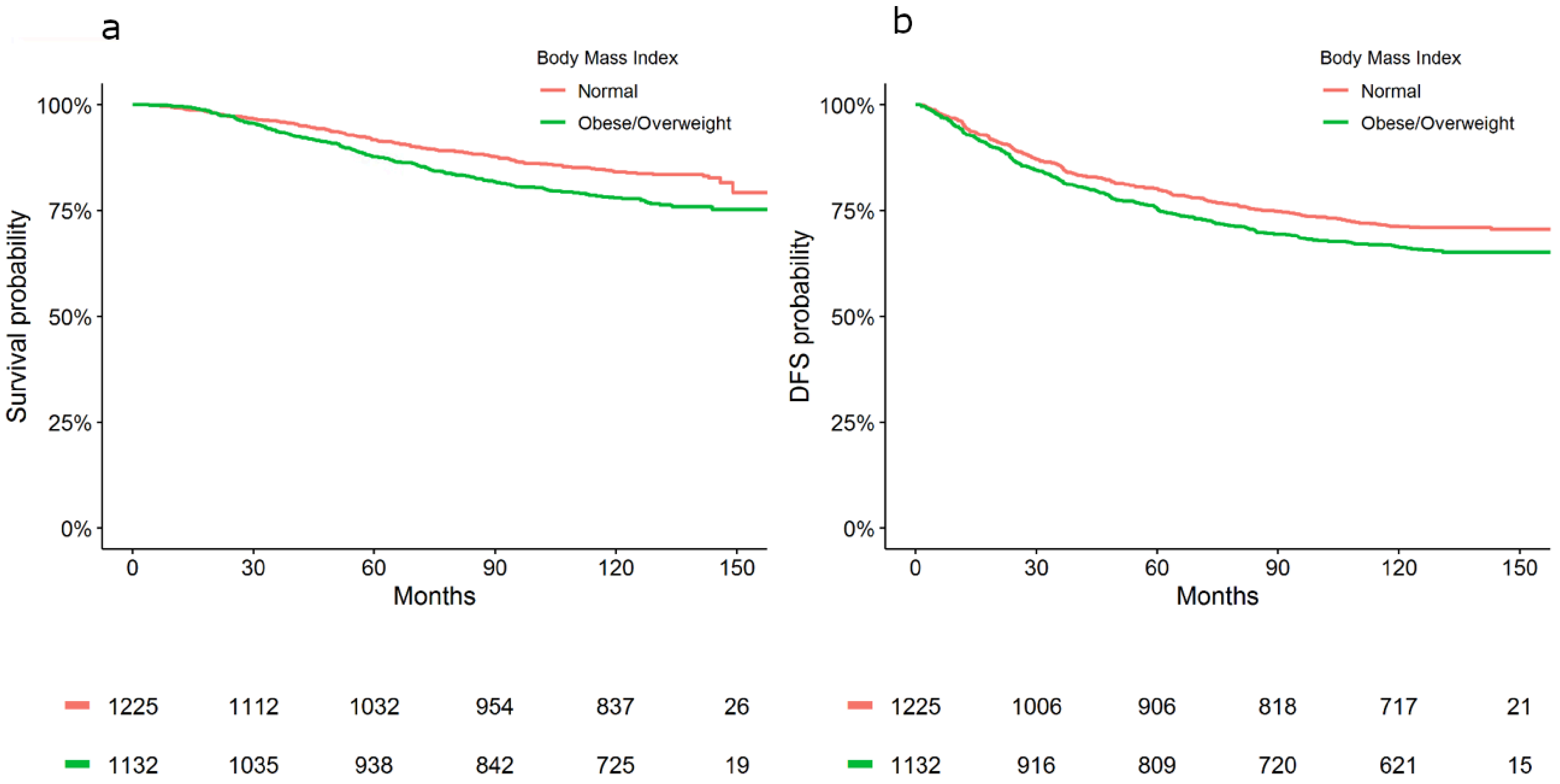

Supplementary Figure 2: Kaplan-Meier plot representing survival outcomes by BMI status in early breast cancer ER and PR Negative Population [Data from HERA study] a) Overall survival; b) Disease-free survival

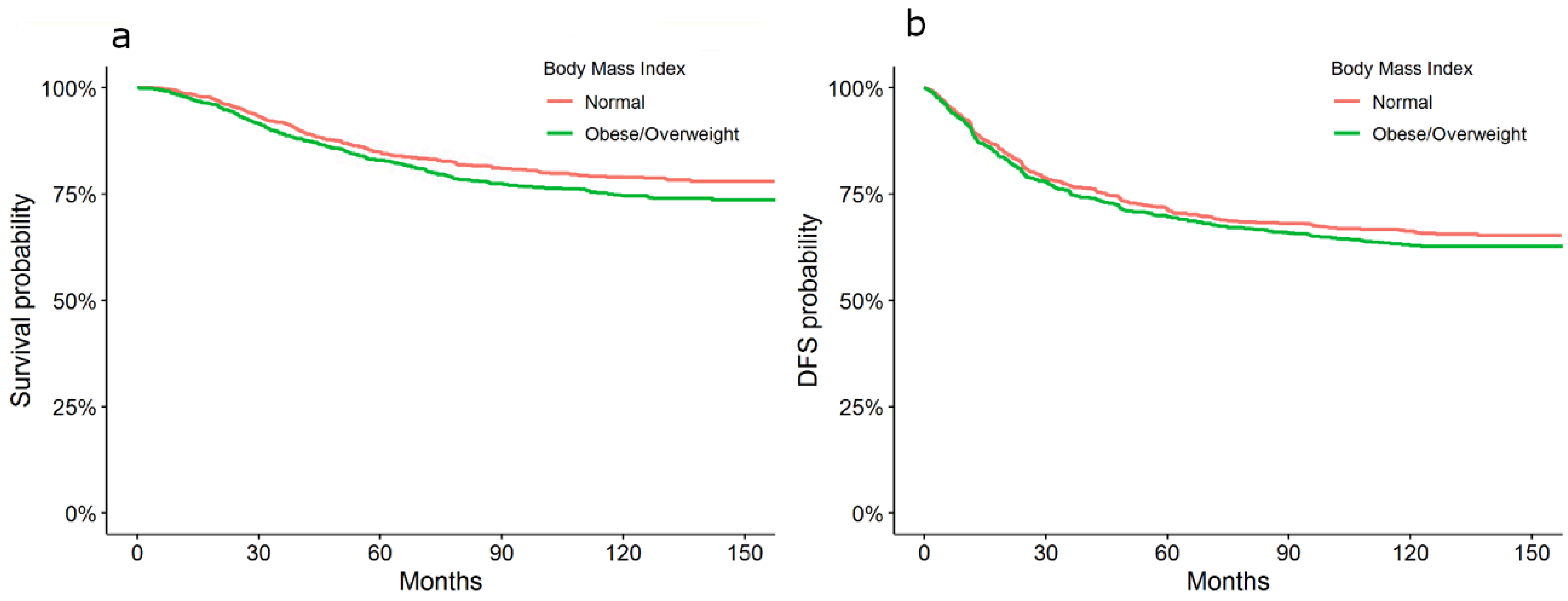

Normal	1116	987	868	795	709	14	Normal	1116	840	734	670	597	14
Obese/Overweight	1258	1099	975	872	769	19	Obese/Overweight	1258	943	825	746	657	18

Supplementary Figure 3: Kaplan-Meier plot representing survival outcomes by BMI status in advanced breast cancer ER and PR Negative Population. a) Overall survival [Data from CLEOPATRA & MARIANNE study]; b) Overall survival [Data from EMILIA & TH3RESA study]; c) Progression-free survival [Data from CLEOPATRA & MARIANNE study]; d) Progression-free survival [Data from EMILIA & TH3RESA study]

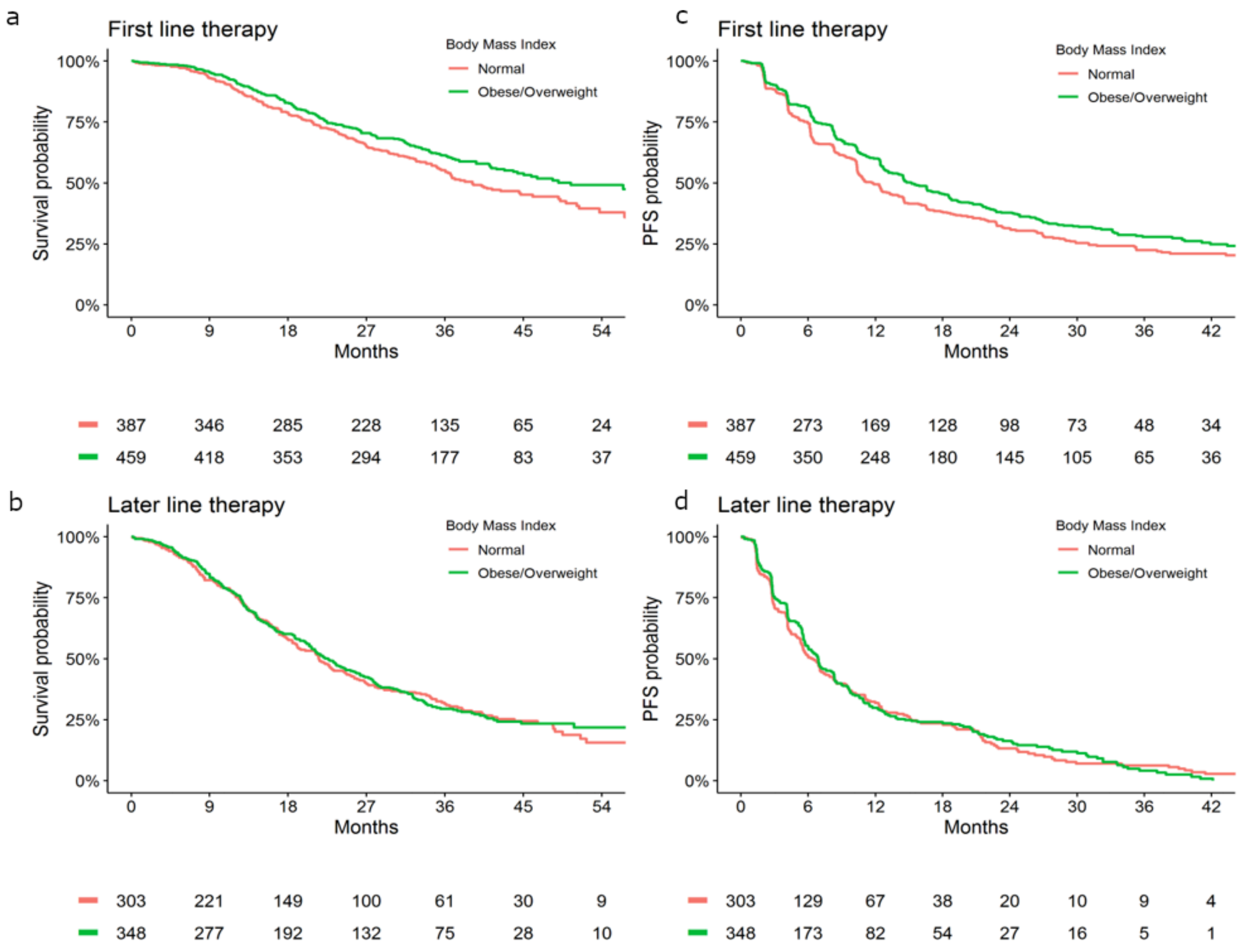

Supplementary Figure 4: Kaplan-Meier plot representing survival outcomes by BMI status in advanced breast cancer ER/PR positive population. a) Overall survival [Data from CLEOPATRA & MARIANNE study]; b) Overall survival [Data from EMILIA & TH3RESA study]; c) Progression-free survival [Data from CLEOPATRA & MARIANNE study]; d) Progression-free survival [Data from EMILIA & TH3RESA study]

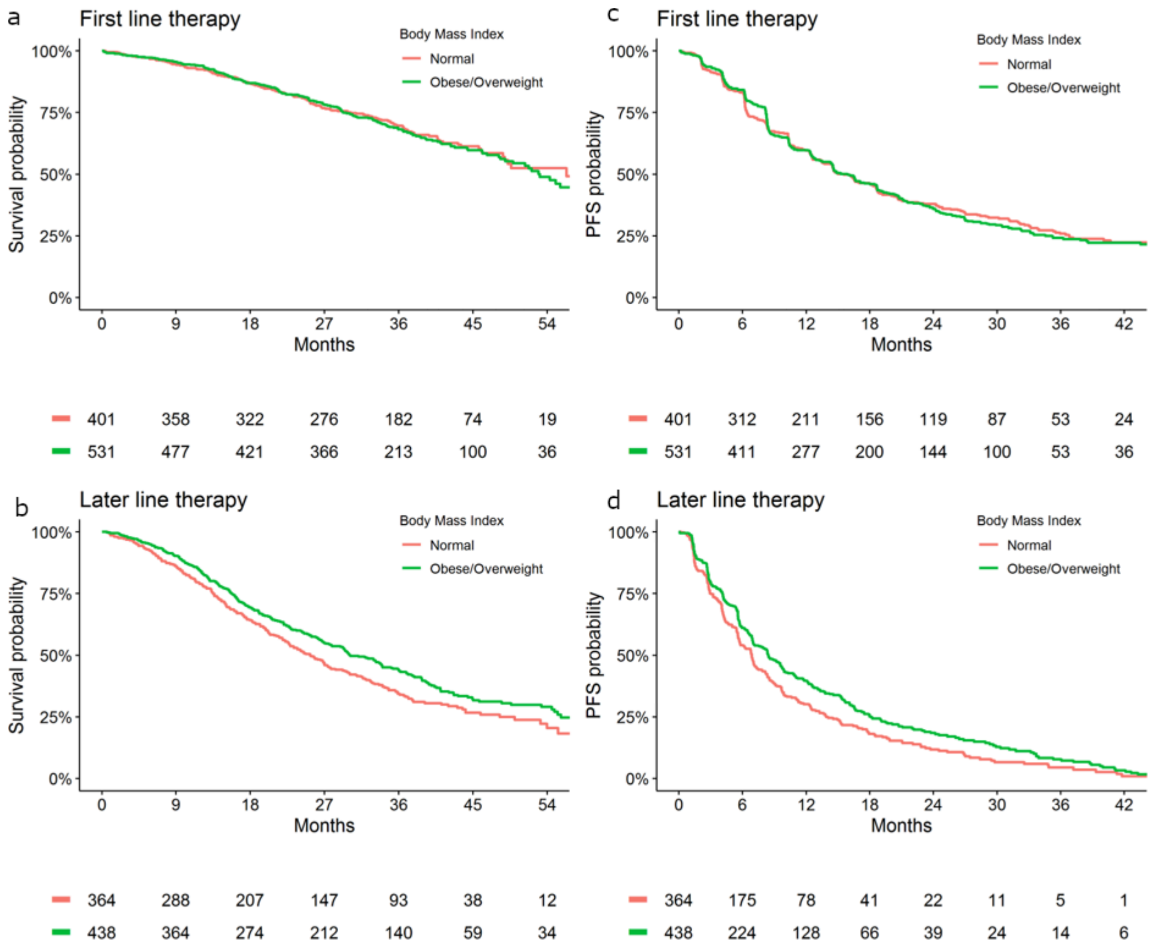

Supplement: Supplementary file 1 — Supplementary Tables and Figures [file 41523_2021_241_MOESM1_ESM.pdf]
